# Elevated Plasma Histone H4 Levels Are an Important Risk Factor in the Development of Septic Cardiomyopathy

**DOI:** 10.4274/balkanmedj.galenos.2019.2019.8.40

**Published:** 2020-02-28

**Authors:** Nian-Fang Lu, Li Jiang, Bo Zhu, De-Gang Yang, Rui-Qiang Zheng, Jun Shao, Jing Yuan, Xiu-Ming Xi

**Affiliations:** 1 Clinic of Critical Care Medicine, Beijing Electric Power Hospital, Beijing, China; 2 Department of Critical Care Medicine, Capital Medical University Fuxing Hospital, Beijing, China; 3 Department of Spinal and Neural Functional Reconstruction, China Rehabilitation Research Center, School of Rehabilitation Medicine, Capital Medical University, Beijing, China; 4 Clinic of Critical Care Medicine, Subei People’s Hospital of Jiangsu Province, Jiangsu, China; 5 Clinic of Cardiac Function Tests, Subei People’s Hospital of Jiangsu Province, Jiangsu, China

**Keywords:** Cardiac troponin I, histones, risk factors, sepsis, septic cardiomyopathy

## Abstract

**Background:**

Myocardial impairment is a major complication and an important prognostic predictor of sepsis. Therefore, early and accurate diagnosis as well as timely management of septic cardiomyopathy is critical to achieve favorable outcomes.

**Aims:**

To investigate the risk factors of septic cardiomyopathy.

**Study Design:**

Cross-sectional study.

**Methods:**

This study performed between May 2016 and June 2018 recruited 93 septic patients from the intensive care unit. All patients received standardized treatments. Septic patients were divided into two groups: non cardiomyopathy (n=45) and septic cardiomyopathy group (n=48). Blood samples were collected and transthoracic echocardiography was performed within 24 hours of intensive care unit admission. Septic patients with one ultrasound abnormality but no history of heart disease were diagnosed as having septic cardiomyopathy. Plasma histones, cardiac troponin I, and N-terminal pro-brain natriuretic peptide were measured using ELISA. Sequential Organ Failure Assessment scores, vasopressor use, and the outcomes of intensive care unit stay were analyzed. Spearman rank analysis was used to determine the correlation between plasma histone H4 and other parameters. Binary logistic regression and receiver operating characteristic curve analysis were used to determine the risk factors for septic cardiomyopathy.

**Results:**

Compared with the non-cardiomyopathy group, the septic cardiomyopathy group had significantly higher plasma H4 and cardiac troponin I levels, a higher Sequential Organ Failure Assessment score, more frequent vasopressor use, and a higher mortality rate (p<0.05). Plasma histone H4 levels positively correlated with cardiac troponin I (r=0.577, p<0.001), N-terminal pro-brain natriuretic peptide (r=0.349, p=0.001), and Sequential Organ Failure Assessment scores (r=0.469, p<0.001). Binary logistic regression and receiver operating characteristic curve analyses revealed that elevated plasma histone H4 levels and vasopressor use were important risk factors for septic cardiomyopathy (p<0.05).

**Conclusion:**

Elevated plasma histone H4 levels could be used to predict septic cardiomyopathy in patients with sepsis.

Sepsis is a life-threatening condition caused by uncontrolled systemic inflammation resulting from infection. Septic shock is a hypotensive condition characterized by elevated blood lactate levels (Lac>2 mmol/L) and cannot be corrected solely by fluid resuscitation (1). Myocardial impairment is a major complication and an important prognostic predictor of sepsis. Approximately 40% of patients with sepsis and septic shock exhibit varying degrees of septic cardiomyopathy (2). In general, approximately 50% of patients with sepsis and septic shock underwent septic cardiomyopathy and once septic cardiomyopathy occurred, the mortality rate of those patients reached from 27% to as high as 70% (3,4). Since myocardial dysfunction caused by septic cardiomyopathy is reversible within 7 to 10 days of its onset, accurate early diagnosis and timely management of septic cardiomyopathy is critical for favorable outcomes (5,6).

Septic cardiomyopathy is primarily assessed using hemodynamic devices such as a pulmonary artery floating catheter (PAC) and pulsed continuous cardiac output (PiCCO) monitoring. However, PAC and PiCCO suffer several drawbacks including an inability to monitor cardiac diastolic function and right heart function, invasiveness, potential catheter-related infection, and difficulty with data measurement and interpretation. Therefore, solely relying on PAC and PiCCO may lead to significant underestimation of the actual incidence of septic cardiomyopathy (7). Echocardiography with tissue Doppler technology has several advantages over PAC and PiCCO. For instance, because it is less likely to be affected by cardiac preload and afterload, tissue Doppler imaging can measure cardiac function more accurately, objectively, and quantitatively (8). Hence, echocardiography has become the gold standard in the detection of septic cardiomyopathy (9).

Histones are a class of proteins that bind to DNA in eukaryotic cells to form chromatin (10). The correlation between plasma histone levels and cardiac function has been previously examined in a mouse model. Kalbitz et al. (11) found that mouse plasma histone levels were positively correlated with myocardial damage. Alhamdi et al. (12) reported that plasma histone levels positively correlated with noradrenaline dose in septic patients but negatively correlated with left ventricular ejection fraction (LVEF). Moreover, administering histone antibodies to septic mice ameliorated cardiac dysfunction (11). These observations suggest that plasma histone levels may be involved in cardiac damage induced by sepsis. Extracellular histones are cytotoxic to endothelial cells. Toxicity is mainly produced by histones H3 and H4 in a dose-dependent manner (13,14,15). Ekaney et al. (14) also found that elevated histone H4 levels were significantly associated with increased mortality in septic patients. However, there is no clinical report about the predictive value of plasma histone H4 in the occurrence of septic cardiomyopathy.

The present study investigated the epidemiological data of patients with septic cardiomyopathy using echocardiography, elucidated the correlation between plasma histone H4 levels and commonly used markers of myocardial injury, and identified the significance of histone H4 in predicting the development of septic cardiomyopathy.

## MATERIALS AND METHODS

### Patient selection

All procedures in this study complied with medical ethical standards and were approved by the Medical Ethics Committee of the hospital (protocol no. 20160223). A total of 158 patients with sepsis who were admitted to the intensive care unit (ICU) of a grade-A first-class general hospital between May 2016 and June 2018 were initially registered. Patients who were diagnosed with sepsis (sepsis=infection + a Sequential Organ Failure Assessment (SOFA) score ≥2) (16) were included in this study. Patients with any of the following conditions were excluded from this study: (1) previous history of chronic heart failure (n=6), (2) localized ventricular wall abnormalities resulting from acute or previous myocardial infarction (n=4), (3) a history of dilated or hypertrophic obstructive myocardium (n=3), (4) previous valvular heart disease (n=6), (5) age <18 years or >80 years (n=12), (6) pregnancy (n=0), (7) atrial fibrillation (interference with cardiac ultrasonography) (n=23), (8) hospitalization time <24 hours (n=1), (9) no echocardiographic data obtained within 24 hours after admission (n=5), or (10) unclear ultrasound images (n=5). After applying the inclusion and exclusion criteria, 56 patients were excluded, and 93 septic patients were included in this study. The study population was divided into two groups: non-cardiomyopathy (n=45) and septic cardiomyopathy (n=48).

### Echocardiography

Routine transthoracic echocardiography (TTE) was performed three times (on the 1^st^, 3^rd^, and 7^th^ day after ICU admission) using a GE Vividi color ultrasound system (USA) with an s3 Rs probe with a f of 1.7 to 3.4 MHz. TTE was operated by an ultrasound physician who was blinded to the patient groups. The following parameters were obtained from TTE: LVEF, peak early diastolic transmitral flow velocity (E), peak late diastolic transmitral flow velocity (A), peak early diastolic mitral annular velocity (e’), left ventricular systolic mitral (LV-Sm) annulus velocity, and right ventricular systolic tricuspid (RV-Sm) annulus velocity. An apical four-chamber view was acquired during the measurement. When the tissue Doppler mode was initiated, the pulsed tissue Doppler sample volume was placed on the side wall of the mitral annulus to obtain the LV-Sm annulus velocity, i.e., the systolic S wave peak velocity. To measure LVEF, we used M-mode ultrasound in patients with normal ventricular wall motion and the Simpson’s method in patients with regional wall motion abnormality. Left ventricular systolic dysfunction was defined as LV-Sm <8 cm/s or LVEF <50%. The pulsed tissue Doppler sample volume was placed on the side wall of the tricuspid annulus to obtain the systolic S peak wave velocity. The right ventricular systolic dysfunction was defined as RV-Sm <12 cm/s. The pulsed tissue Doppler sample volume was placed on the side wall of the mitral annulus to obtain E and e’. The left ventricular diastolic dysfunction was defined as E/e ‘>15 or e’< 8 cm/s.

Septic cardiomyopathy was diagnosed based on the following three criteria: 1) the patient was diagnosed with sepsis, 2) the patient had one of the following three ultrasound abnormalities: A) LV-Sm <8 cm/s or LVEF <50%; B) RV-Sm <12 cm/s; or C) E/e’>15 or e’ <8 cm/s, and 3) the patient had no history of heart disease, including chronic heart failure regional ventricular wall motion abnormality, dilated cardiomyopathy, hypertrophic obstructive cardiomyopathy, or heart valve disease.

### Blood collection and biochemical measurements

Demographic and baseline clinical characteristics were obtained from all participants. These included age, gender, body mass index (BMI), past disease, site of infection, and disease diagnosis. All enrolled patients were administered antibiotics and fluid resuscitation in compliance with treatment guidelines. Vasoactive drugs and ventilators were also used if necessary. Circulating levels of troponin, N-terminal pro-brain natriuretic peptide (NT-proBNP), and histone H4 were measured using ELISA [Human cardiac troponin I (ctnI) ELISA Kit, Human NT-proBNP ELISA Kit, and Human Histone H4 ELISA Kit were purchased from Beijing Leagene Biotech, China]. In brief, blood samples from patients were collected into ethylenediaminetetraacetic acid tubes (Sainty International Group Jiangsu Yangzhou Sumex Imp. & Exp. Co., Ltd. China) within 24 hours of admission to the ICU. Supernatants were obtained after centrifugation at 3,000 rpm for 10 minutes and were frozen at -80° within half an hour for subsequent analysis. The following data were recorded from each participant: blood procalcitonin (PCT), lactate level, SOFA scores, vasopressor use, length of ICU stay (d), and outcomes of ICU stay.

For the histone H4 assays, wells for both standards and tests were prepared. To generate a standard curve, 50 μL of the standard solution was added, while 10 μL of the test sample plus 40 μL of sample diluent were added to the test wells. A blank well was also prepared. Next, 100 μL of an HRP-conjugated reagent was added and incubated for 60 minutes at 37°, followed by four washes with wash solution (400 μL). Thereafter, 3,3′-diaminobenzidine substrate chromogen solution (50 μL) and 3% aquae hydrogenii dioxide substrate chromogen solution B (50 μL) were added to each well and incubated for 15 minutes at 37° in the dark. Using a microtiter plate reader, optical density was measured at a wavelength of 450 nm within 15 minutes of addition of the stop solution (50 μL).

### Statistical analysis

All statistical analyses were performed using the SPSS 22.0 statistical software package. Normally distributed data are presented as mean ± standard deviation (x ± SD) and non-normally distributed data are presented as median (25th percentile, 75th percentile) [M (QL, QU)]. The independent sample t-test was used to compare normally distributed data, while the Wilcoxon rank sum test was used for comparing non-normal data. Count data are expressed as frequency (f), composition ratio, or percentage, and statistically analyzed using the chi-square test. Spearman rank correlation analysis was used to determine the correlation between plasma histone H4 and cTnI and NT-proBNP levels. Binary logistic regression and a receiver operating characteristic (ROC) curve were used to determine the risk factors for prognosis of septic cardiomyopathy. Triplicates were used in each case. The evaluation index is represented as the odds ratios (ORs), and p<0.05 was considered statistically significant.

## RESULTS

### Comparison of demographic and baseline clinical characteristics of patients who survived and those who died

A total of 93 patients, including 71 patients with septic shock and 22 patients without shock, were enrolled in this study. Among these patients, 64 survived whereas 29 died during ICU treatment. There was no significant difference in the number of primary infection sites between the patient group that died versus those that survived (p>0.05) (Table 1). No significant differences in age, gender, and BMI were observed between the two groups (p>0.05). In addition, there were no significant differences in length of ICU stay or plasma levels of NTpro-BNP, lactate, and PCT between the two groups (p>0.05 for all). However, patients who died had a significantly higher SOFA score and more frequent vasopressor use compared to those who survived (p<0.05) (Table 1).

### Distribution of different types of septic cardiomyopathy

**Figure 1:**
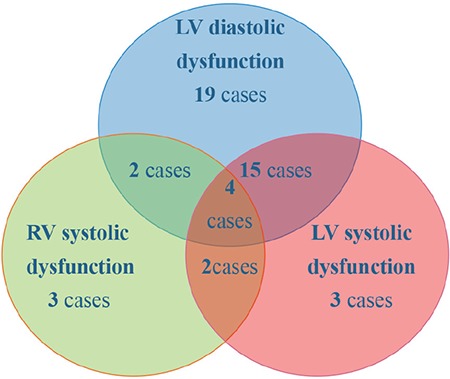
Distribution of different types of septic cardiomyopathy.

Among the 93 patients with sepsis, 40 had left ventricular diastolic dysfunction, 24 had left ventricular systolic cardiomyopathy, and 11 had right ventricular systolic dysfunction. Of those with cardiomyopathy, 19 suffered concomitantly from left ventricular systolic and diastolic dysfunctional cardiomyopathy, and 4 concomitantly suffered from left ventricular systolic, diastolic, and right ventricular dysfunction. A total of 48 patients (51.6%) had septic cardiomyopathy (Figure 1).

### Correlation between plasma histone H4 levels and cTnI, NT-proBNP, and SOFA score

**Figure 2:**
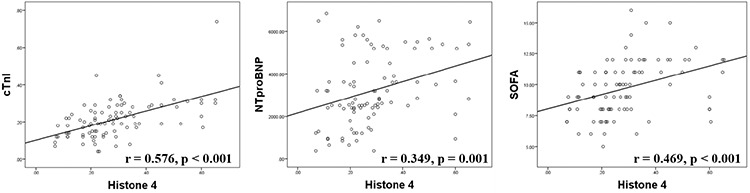
Correlation between plasma histone H4 and other parameters. NT-proBNP: N-terminal pro-brain natriuretic peptide, SOFA: sequential organ failure assessment

We used the Spearman correlation analysis to determine the correlation between plasma histone H4 levels and cTnI, NT-proBNP, and SOFA score. As shown in Table 2, plasma histone H4 levels strongly correlated with cTnI (r=0.577, p<0.001) and SOFA score (r=0.469, p<0.001) and had a weak but positive correlation with NT-proBNP (r=0.349, p=0.001) (Table 2, Figure 2).

### Comparison of demographic and baseline clinical characteristics between non-cardiomyopathy and septic cardiomyopathy groups

As shown in Table 3, the septic cardiomyopathy group had a significantly higher SOFA score and mortality rate, elevated plasma histone H4 levels and cTnI levels, and more frequent vasopressor use compared to the non-cardiomyopathy group (p<0.05). There were no statistical differences in age, gender, BMI, plasma NT-pro-BNP, and baseline characteristics between the two groups. (p>0.05) (Table 3).

### Determination of risk factors of septic cardiomyopathy

**Figure 3:**
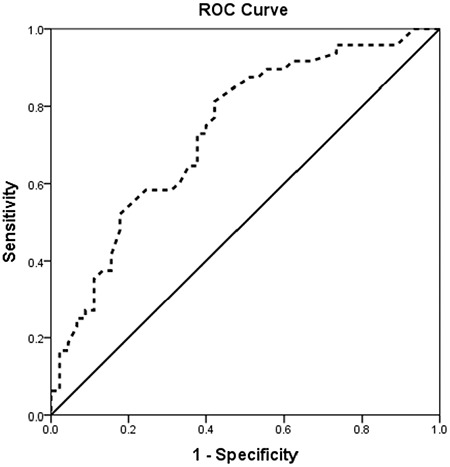
ROC curve analysis of plasma histone H4 for predicting the development of cardiomyopathy in patients with sepsis. At the cutoff point, the sensitivity and specificity for plasma histone H4 were significant. ROC: receiver operating characteristic

Binary logistic regression analysis was used to determine the risk factors for septic cardiomyopathy. SOFA score, vasopressor use, plasma histone H4 levels, and cTnI levels were included in the binary logistic regression analysis. As shown in Table 4, we found that frequent vasopressor use (p=0.004, OR=7.514) and elevated histone H4 levels (p=0.016, OR=21.5) were significant predictors of septic cardiomyopathy. ROC curve analysis showed that histone H4 level of 0.22 ug/mL could predict septic cardiomyopathy with a sensitivity of 81.3%, specificity of 57.8%, AUC of 0.734 (p<0.05), positive predictive value (PPV) of 64.6%, and a negative predictive value (NPV) of 78.6% (Figure 3).

## DISCUSSION

In the present study, we investigated the risk factors of septic cardiomyopathy and found that: 1) sepsis patients who died in the ICU had significantly higher SOFA scores and more frequent vasopressor use, 2) patients in the septic cardiomyopathy group had significantly higher plasma histone H4 levels, cTnI levels, and more frequent vasopressor use, and 3) elevated plasma histone H4 levels and frequent vasopressor use were important risk factors for septic cardiomyopathy.

### Distribution of different types of septic cardiomyopathy

Previously, septic cardiomyopathy substantially increased the mortality rate (up to 70%) in septic patients (3,16,17). In this study, the incidence of septic cardiomyopathy was 51.6%, and the mortality rate was 41.7%. Lower morality rate in our study compared to previous studies is likely due to the small sample size of our study. Previous studies also showed that septic cardiomyopathy was not limited to the left heart, as the right heart was often affected (18,19). In line with these findings, we found that the highest incidence of cardiac dysfunction in septic cardiomyopathy was due to left ventricular diastolic dysfunction (43%), followed by left ventricular systolic cardiomyopathy (25.8%), and right ventricular systolic dysfunction (11.8%). In addition, different types of cardiomyopathy may occur simultaneously. For instance, 8.3% of septic cardiomyopathy patients in our study had combined left ventricular systolic, diastolic, and right ventricular dysfunction.

### Elevated plasma histone H4 levels in patients with sepsis and septic cardiomyopathy

Histones are structural proteins in eukaryotic nuclei that bind to DNA and form nucleosomes, the basic structural unit of chromatin (10). Under physiological conditions, circulating blood contains extremely low levels of nucleosomes (20). However, during the final stage of apoptosis, nucleosomes are released from cells into the blood (21,22), elevating circulating histone levels. Histones are released from the nucleus in response to infection, inflammation, microvascular thrombosis, and endothelial cell dysfunction and play an important role in the pathogenesis of sepsis (13,23). Previous findings also pointed to a potential role of plasma histones in the pathogenesis of septic cardiomyopathy (11). Possible mechanisms may include histone-mediated calcium influx, loss of homeostasis in the redox system, and defects in mitochondria in cardiomyocytes (11). In the present study, we found that plasma histone H4 levels in patients with septic cardiomyopathy were significantly higher than the levels in septic patients with no cardiomyopathy. It is likely that sepsis patients with significantly elevated histone H4 levels may exhibit more severe cardiomyocyte damage. This potential feedback loop may further exacerbate cardiac dysfunction.

### Correlation between plasma histone H4 levels and cTnI and NT-proBNP in patients with septic cardiomyopathy

cTnI and NT-proBNP are the two most commonly used clinical markers of myocardial injury. cTnI can only be released into the blood when the myocardial cell membranes are injured and is highly sensitive and specific for myocardial injury (24). Favory et al. (25) reported that increased plasma cTnI levels induced by sepsis were caused by multifactorial-linked minimally invasive myocardial damage during the progression of sepsis. A more recent study has shown that sepsis-induced elevation in circulating histones was associated with cardiomyocyte damage, which resulted in elevated plasma cTnI levels (26). In addition, in patients with septic shock, cTnI levels were elevated and strongly correlated with cardiac dysfunction as evidenced by echocardiography (27). It was previously reported that there is a positive correlation between circulating histone levels and cTnI (12). Indeed, our findings support the hypothesis that circulating levels of histones correlate with plasma cTnI in septic cardiomyopathy.

NT-proBNP is a neuroendocrine hormone that is mainly synthesized in and secreted from ventricular myocytes and is released in large amounts as ventricular wall tension increases. A previous study (28) showed that plasma NT-proBNP levels correlated with the presence of septic cardiomyopathy, as well as the prognosis of septic patients. Parker et al. (29) found that the combination of acute ventricular dilatation, ventricular pressure load, and volume overload was an important mechanism leading to NT-proBNP release. In addition, both lipopolysaccharide and inflammatory factors have been shown to elevate NT-proBNP levels in sepsis (30). In this study, we found that blood histone H4 and NT-proBNP levels were weakly correlated compared to the strong association between histone H4 levels and cTnI. This may be because increase in NT-proBNP is mainly associated with increased ventricular wall tension, while the increase in circulating histones is mainly injury-related.

Historically, there was insufficient evidence to support the standard use of troponin or pro-BNP as a biomarker for septic cardiomyopathy. We provide evidence of an additional biomarker, histone H4, in predicting septic cardiomyopathy. Nonetheless, identification of other novel biomarkers is encouraged.

### Risk factors for septic cardiomyopathy

Few studies have examined the independent risk factors for septic cardiomyopathy. In this study, we found that plasma histone H4 levels and vasopressor use were both important risk factors for the development of septic cardiomyopathy. An OR of 21.5 for histone H4 meant that the probability of septic cardiomyopathy increased 20.5 times, while the value of histone H4 increased one unit, suggesting the key role of histone H4 in predicting the development of septic cardiomyopathy. ROC analysis showed moderate accuracy of H4 as a predictor of septic cardiomyopathy as its NPV was higher than its PPV. Previously, Kalbitz et al. (11) found that plasma histone levels caused an imbalance of intracellular calcium ion levels and redox reactions in mouse cardiomyocytes, leading to myocardial damage and eventual cardiac dysfunction. Accordingly, a histone antibody partially attenuated the sepsis-induced myocardial damage in a mouse model (11). Fattahi et al. (31) also reported that elevated extracellular histone levels were an important contributor to the development of septic cardiomyopathy. These findings collectively suggest an important pathogenic role for histones in septic cardiomyopathy. In agreement with the above observations, we found that blood histone H4 levels were positively correlated with plasma cTnI concentrations and SOFA score. The SOFA score is one of the most commonly used scoring systems to clinically determine the severity of sepsis in critically ill patients. Higher SOFA score is associated with more severe disease (32). Indeed, we noted that patients with septic cardiomyopathy had a higher SOFA score, which was associated with higher levels of plasma histones H4 and cTnI. Another interesting finding from this study was that vasopressor use was an independent risk factor for developing septic cardiomyopathy.

In septic shock, microcirculatory disorders, abnormal blood flow distribution, and an imbalance between oxygen supply and demand affect cardiac perfusion. This results in myocardial hypoxia, which may be another important cause of septic cardiomyopathy. Therefore, our findings indicate that severe sepsis and/or septic shock are indicators of vasopressor use. We noted that septic shock was accompanied by extremely low blood pressure requiring medication (33). Thus, blood pressure treatment for septic shock patients should be personalized to achieve the best clinical outcome (34,35).

Some limitations of this study need to be acknowledged. Firstly, although this was a prospective study, we did not perform a cause-effect investigation. Secondly, as the study endpoint was patient outcomes during ICU stay, it did not include long-term follow-up analysis. Thirdly, this was a single-center study with a small sample size. Multi-centered large cohort studies are needed to further corroborate our findings. Lastly, we acknowledge that the accuracy and reproducibility of ELISA in measuring plasma histone levels is less than satisfactory and a more efficient and sensitive tool is needed for histone identification and quantification.

In conclusion, the current study demonstrates that elevated plasma histone H4 levels could potentially be used as a predictive biomarker for cardiomyopathy in patients with sepsis.

## Figures and Tables

**Table 1:**
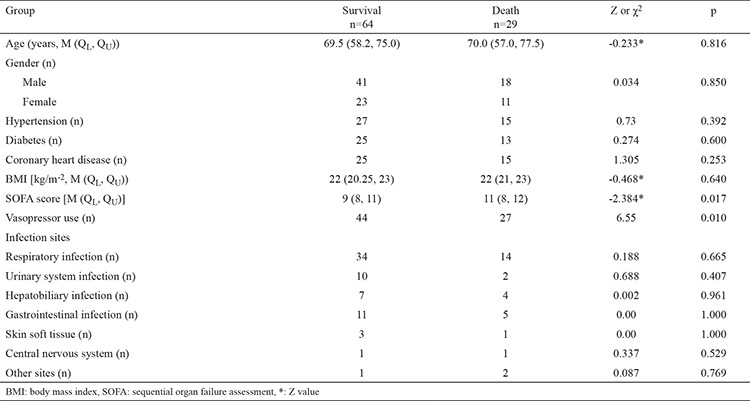
Comparison of demographic and baseline clinical characteristics of patients between survival and death groups

**Table 2:**
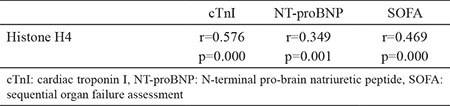
Correlation analysis of plasma histone H4 and various parameters

**Table 3:**
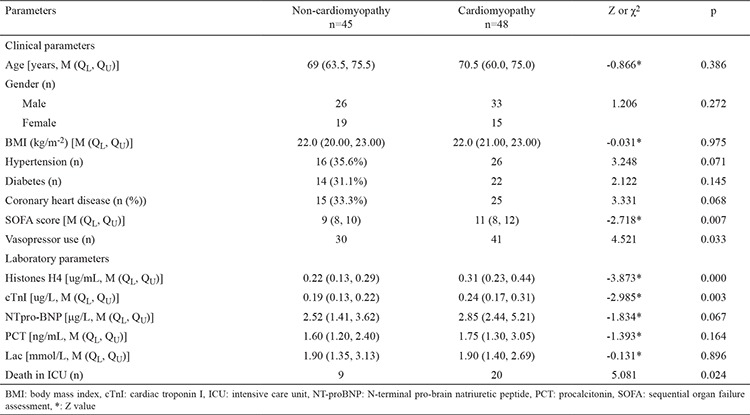
Comparison of demographic and baseline clinical characteristics between the non-cardiomyopathy and septic cardiomyopathy groups

**Table 4:**
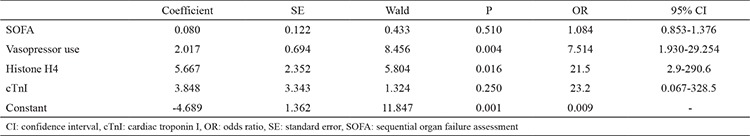
Determination of risk factors for septic cardiomyopathy
